# Biopreservation of Ground Beef Patties Using Lactic Acid: A Sustainable Alternative to Synthetic Additives

**DOI:** 10.1155/ijfo/9930525

**Published:** 2025-11-21

**Authors:** Michel M. Beya, Michael E. Netzel, Yasmina Sultanbawa, Heather E. Smyth, Louwrens C. Hoffman

**Affiliations:** ^1^ Centre for Nutrition and Food Sciences, Queensland Alliance for Agriculture and Food Innovation, The University of Queensland, Brisbane, Queensland, Australia, uq.edu.au; ^2^ ARC Industrial Transformation Training Centre for Uniquely Australian Foods, Queensland Alliance for Agriculture and Food Innovation, The University of Queensland, Brisbane, Queensland, Australia, uq.edu.au

**Keywords:** antimicrobial, antioxidant, biopreservative, burger patties, lactic acid

## Abstract

The use of synthetic chemicals in meat products has raised consumer health concerns, driving increased interest in natural preservatives. This study evaluated the effectiveness of lactic acid (LA) as a natural preservative in raw beef patties, comparing it to sodium metabisulphite (SMB; 450 ppm) and a negative control (NC). Ground beef was treated with varying concentrations of LA (0.125%, 0.25%, 0.375% and 0.5%) and stored at 4^°^C ± 1^°^C for 20 days. Microbial growth, lipid oxidation, pH, colour, cooking yield and texture were analysed throughout the storage period. Results showed that LA‐treated patties significantly inhibited microbial growth (*p* < 0.05) compared to NC, though SMB exhibited the strongest antimicrobial effect. Lipid oxidation levels in LA‐treated patties were comparable to NC (*p* > 0.05), whereas SMB‐treated patties had the lowest oxidation values (*p* < 0.05). Increasing LA concentrations led to a reduction in pH, whilst colour analysis revealed decreased redness and higher metmyoglobin content in LA‐treated samples. Texture profile analysis showed no significant differences in hardness, cohesiveness, gumminess or chewiness amongst treatments; however, springiness was affected (*p* < 0.05). These findings suggest that LA, at concentrations of 0.125% or higher, can effectively extend shelf life, offering a natural alternative to synthetic preservatives like SMB whilst aligning with consumer preferences for cleaner label meat products.

## 1. Introduction

Over the past two decades, global meat consumption has steadily increased, reaching 328.4 million tonnes in 2021 [1]. Processed meat, which accounts for approximately 44% of total meat consumption, undergoes extensive handling during production, making it more susceptible to safety and quality degradation [2]. The shelf life of meat and meat products largely depends on factors such as the initial microbial load, adherence to good manufacturing practices, packaging methods and storage conditions [3]. Additionally, processing techniques such as chopping and mincing increase the meat′s exposure to oxygen, which accelerates lipid oxidation. This process leads to the degradation of essential fatty acids, flavour loss and discoloration, ultimately affecting organoleptic properties and reducing consumer acceptability [4]. Microbial contamination and lipid oxidation are the two primary factors contributing to the reduced shelf life and food safety risks associated with ground meats, including beef patties. Addressing these challenges is crucial for maintaining product quality and ensuring consumer safety.

To mitigate quality deterioration, enhance safety and extend the shelf life of meat products, synthetic preservatives are commonly used. Synthetic antioxidants such as sodium lactate, ethoxyquin, tocopherols, tert‐butylhydroquinone (TBHQ), butylated hydroxytoluene (BHT), butylated hydroxyanisole (BHA) and gallates are widely employed to delay oxidative reactions in ground beef [5]. Additionally, synthetic antimicrobials such as sulphites, nitrites, phosphates, potassium sorbate, propylparaben, sodium benzoate, sodium methyl, sodium diacetate, acidified sodium chloride, acidified calcium sulphate and methylpyridinium chloride are frequently used to inhibit microbial growth in meat products [5]. However, growing concerns regarding the safety of these synthetic preservatives amongst health authorities and consumers have led to stricter regulations on their use in food formulations [6]. As a result, there has been a significant shift in interest towards naturally occurring preservatives as safer and more consumer‐friendly alternatives.

The use of natural preservatives, such as lactic acid (LA), is gaining popularity due to increasing concerns over the safety of synthetic preservatives. LA (L‐LA) is a naturally occurring C_3_ *α*‐hydroxycarboxylic acid and a byproduct of sugar metabolism by microorganisms under anaerobic conditions [7]. It has diverse applications across various industries, including food, textiles, leather, cosmetics and pharmaceuticals [8]. Several studies have demonstrated that organic acids, including LA, effectively inhibit pathogen growth on beef carcasses and cuts. However, their effectiveness as decontaminants for beef trims has yielded inconsistent results, highlighting the need for further investigation [9, 10].

Previous studies showed that incorporating food‐grade LA into beef formulations can enhance storage stability and quality attributes without compromising sensory acceptability. For example, [11] carried out a study using 0.5%–1.0% LA; beef patties exhibited reduced pH, lower microbial counts and greater physicochemical stability compared with untreated controls during 12 days of refrigerated storage. Importantly, LA incorporation did not significantly affect water activity or overall consumer acceptability, although treated patties showed lower hardness. These findings highlight the potential of LA as a practical preservative to extend shelf life in fresh beef products whilst maintaining desirable quality. Furthermore, other studies have shown that LA bacteria, through the production of LA, can exhibit antioxidant properties, helping to prevent the oxidation of fats and other compounds in meat [12, 13]. Oxidation is a significant challenge in the meat industry, as it can lead to undesirable effects such as off‐flavours, discoloration and spoilage [14].

In this study, beef patties were used as a model system due to their widespread popularity and high susceptibility to safety and quality degradation. The primary objective was to evaluate the efficacy of LA as a natural preservative. The findings of this study provide valuable insights into the effectiveness of LA in preserving beef products and potentially other meat products. Additionally, this research contributes to the broader understanding of effective preservation strategies aimed at enhancing the safety and quality of meat products. The novelty of this study lies in systematically incorporating LA into beef patty formulations at concentrations up to 0.5% in combination under modified atmosphere packaging (MAP). Whilst LA has been widely studied, few investigations have examined its effectiveness at such low incorporation levels under MAP conditions in minced meat systems. By exploring this synergy across concentration gradients, our research provides new insights into optimising preservative strategies that extend shelf life, enhance microbial stability and maintain the sensory and quality attributes of fresh beef products.

## 2. Materials and Methods

### 2.1. Reagents

LA (88%) (food grade) was sourced from Earlee Products Pty Ltd (Murarrie, Queensland, Australia). Analytical‐grade 2‐thiobarbituric acid (TBA), 2,2‐diphenylpicrylhydrazyl (DPPH), 1,1,3,3‐tetramethoxypropane (TMP), methanol, ethanol, gallic acid, Folin–Ciocalteu reagent and phosphate‐buffered saline (PBS) were obtained from either Sigma‐Aldrich or Merck (Macquarie Park, New South Wales, Australia).

### 2.2. Beef Patty Manufacturing and Experimental Design

**Figure 1:**
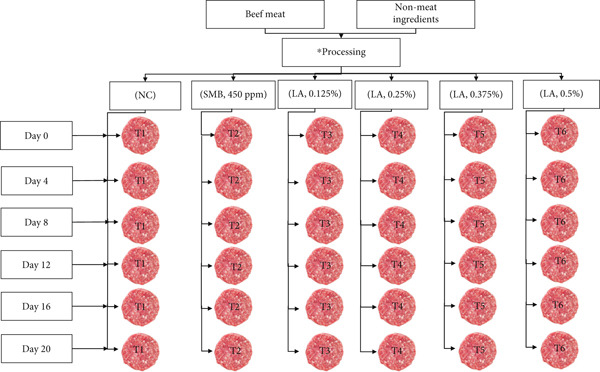
Schematic representation of the experimental design. *The processing includes trimming, weighing, grinding and mixing of ingredients. Three replicates made independently of each other. T (Treatments 1, 2, 3, 4, 5 and 6), NC (negative control), SMB (sodium metabisulphite, 450 ppm), LA (lactic acid) (0.125%, 0.25%, 0.375% and 0.5%). The beef patties were stored at 4^°^C ± 1^°^C for 20 days. Three replicates of each treatment were evaluated [15].

A flow diagram of the sample preparation is shown in Figure 1. This experiment is aimed at producing beef patties using different ingredients and preservatives, with three independent replicates conducted. The meat and dry ingredients were sourced from Australian Country Choice (ACC) (Cannon Hill, Queensland, Australia) and Earlee Products Pty Ltd, respectively. Beef patty processing took place in a pilot plant at ACC. Chilled meat (24 h postmortem; < 2°C) was trimmed to remove excess fat and connective tissue before being cut into uniformly sized chunks. The dry ingredients, including ice/water (7.5%), salt (NaCl; 2%) and potassium polyphosphate (0.5%), were then combined with the meat (90%) as per the basal recipe outlined in Table 1. The mixture was thoroughly hand‐mixed for approximately 5 min before undergoing a two‐step grinding process: an initial grind through a 12‐mm steel plate, followed by a second grind through an 8‐mm steel plate, resulting in finely minced meat.

**Table 1:** Formulation of beef patty treated with lactic acid.

**Ingredients (%)**	**Treatments**					
	**NC**	**SMB (450 ppm)**	**LA 0.125%**	**LA 0.25%**	**LA 0.375%**	**LA 0.5%**
*Basal recipe*						
Meat (90CL) (%)	90	90	90	90	90	90
Water/ice (%)	7.5	7.5	7.5	7.5	7.5	7.5
Salt (NaCl) (%)	2	2	2	2	2	2
Polyphosphate (%)	0.5	0.5	0.5	0.5	0.5	0.5
Total	100	100	100	100	100	100
*Treatments*						
LA (%)	0	—	0.125	0.25	0.375	0.5
SMB (ppm)	—	450	—	—	—	—

*Note:* Basal recipe (meat, water, salt and polyphosphate).

Abbreviations: LA, lactic acid (0.125, 0.25, 0.375 and 0.5) % of the basal recipe; NC, negative control; SMB, sodium metabisulphite (450 ppm).

The minced meat was evenly divided into six subgroups based on weight, with each subgroup assigned to one of six treatments. These treatments included a negative control (NC) sample with no preservative, a sample treated with sodium metabisulphite (SMB; 450 ppm) and four samples containing increasing concentrations of LA at 0.125%, 0.25%, 0.375% and 0.5% of the base recipe. The choice to cap the highest concentration at 0.5% was informed by previous work, such as Al‐Dalali et al. [16], who reported effective preservation outcomes at ≥ 0.5%. Building on this, the present study focused on lower incorporation levels (≤ 0.5%) to evaluate their efficacy under MAP conditions. A total of 36 beef patty samples, each weighing approximately 100 g, were shaped using a plastic burger patty maker. The formed patties were placed in clear polypropylene barrier trays (133 × 224 × 50 mm) and packaged under an MAP containing 80% O_2_ and 20% CO_2_. The trays were then heat‐sealed with a clear antifog barrier shrink film suitable for gas mixtures. This entire procedure was repeated on 3 separate days to obtain three independent replicates.

The packaging film used in this study was composed of polyethylene terephthalate, ethylene vinyl alcohol and polyethylene, with an oxygen transmission rate (OTR) of 2.64 ± 0.12 cc/m^2^/day, a carbon dioxide transmission rate (CDTR) of 2.5 ± 0 cc/m^2^/day, a water vapour transmission rate (WVTR) of 0.69 ± 0.01 g/m^2^/day and a thickness of 90 *μ*m. The sealed trays were transported under chilled conditions and stored in a cold room at 4^°^C ± 1^°^C for a 20‐day shelf‐life study at the Centre for Nutrition and Food Sciences, University of Queensland. During the storage period, samples from each treatment and replicate were collected on Days 0, 4, 8, 12, 16 and 20 for microbiological and physicochemical analysis.

### 2.3. Microbiological Analysis

The total viable count (TVC) of the beef patties was determined following the Australian Standard AS5013.5:2016 for microbiological enumeration [17]. Briefly, a 10 g aliquot of beef patty was aseptically blended with 90 mL of 0.1% sterile peptone water using a Stomacher (400 Seward, London, England) for 2 min. A 1:10 serial dilution (beef patty:peptone water) was then prepared using 9 mL of 0.1% peptone water. The diluted samples were aseptically plated in duplicate. Approximately 12–15 mL of plate count agar (maintained at 44°C–47°C) were poured into each petri plate, followed by gentle rotation to ensure even mixing of the inoculum and the medium. The plates were left undisturbed under aseptic conditions until the agar solidified. Once set, the plates were inverted and incubated at 30^°^C ± 1^°^C for 72 ± 3 h. Results were expressed as log_10_ colony‐forming units (CFU) per gramme of meat.

### 2.4. Lipid Oxidation Analysis

Lipid oxidation in beef patties was assessed by measuring the concentration of malondialdehyde (MDA) formed during storage. The thiobarbituric acid reactive substance (TBARS) assay, with slight modifications from the method described by Mukumbo et al. [18], was used for this analysis.

Briefly, 2 g of each beef patty sample was blended with 12.5 mL of trichloroacetic acid (TCA) (20% in 1.6% metaphosphoric acid) and 12.5 mL of distilled water using a Stomacher 400 Laboratory Blender (Seward Medical, London, United Kingdom) at 260 rpm for 2 min. The homogenate was then filtered through Whatman Grade 2 filter paper. Duplicate 3 mL aliquots of the filtrate were mixed with an equal volume of 0.02 M TBA, whilst a third aliquot was combined with an equivalent volume of distilled water to serve as a turbidity blank.

All samples were vortexed for 30 s using a Vortex‐Genie 2 (Scientific Industries Inc., Bohemia, New York, United States) and incubated in a water bath at 100°C for 35 min. After incubation, the samples were cooled on ice for 5 min, and absorbance was measured at 532 nm using a UV spectrophotometer (Thermo Scientific GENESYS 50, Thermo Fisher Scientific Inc., Rochester, New York, United States). MDA concentration in the samples was determined using a standard curve prepared from serial dilutions of a TMP solution. The TBARS values were expressed as milligramme MDA per kilogramme of the sample, calculated using the following equation:

MDA content=OD sample−OD turbidity blankslope×MMDA×12.103−×1msample g

where OD is the optical density at 532 nm, slope is the slope of the standard curve of the TMP used as standard, *M*_MDA_ is the molecular weight (72.06 g per mol) and *m*_sample_ is the mass of the sample expressed in gramme. Results were expressed as milligramme MDA equivalents (eq) per kilogramme of meat.

### 2.5. pH Value Measurement

The pH measurement was conducted to determine the pH level of the beef patty samples using a digital FC2022 HALO pH metre (Hanna Instruments, Woonsocket, United States). For accuracy, the instrument was calibrated with standard buffer solutions at pH 4.0 and pH 7.0 before measurement. The probe was inserted at two different locations in each beef patty sample, and the average of the two readings was used for statistical analysis.

### 2.6. Colour Parameters and Surface Myoglobin Redox Forms

The colour of beef patties was analysed using a portable spectrophotometer (Model 6834, BYK‐Gardner GmbH, Geretsried, Germany) in accordance with the guidelines for meat colour evaluation [19]. The instrument settings were configured as follows: an 8 mm diameter aperture, illuminant D65 (artificial) and a 10° standard observer angle. Calibration was performed according to the manufacturer′s instructions. Colour measurements were recorded for CIE *L*∗ (lightness), *a*∗ (redness) and *b*∗ (yellowness) on the surface of each beef patty at specific sampling intervals (Days 0, 4, 8, 12, 16 and 20). Each sample was scanned at three different points, and the average of these readings was used for statistical analysis. Additionally, the *C*∗[(*a*∗+*b*∗)1/2] and hue angles [arctan (*b*∗/*a*∗)] were also calculated for each beef patty reading [19]. The surface myoglobin redox forms were determined following the method described by [20].

### 2.7. Surface Myoglobin Redox Forms

The oxymyoglobin (OMb) and metmyoglobin (MtMb) content in ground beef patties was assessed following the guidelines outlined by [19] using a GYK colour guide spectrophotometer (BYK‐Gardner USA, Columbia, United States). Reflectance measurements were taken from each sample at key isosbestic wavelengths of myoglobin: 743, 525, 572 and 730 nm, immediately after opening the patty packaging. Due to the measurement limitations of the GYK colour guide spectrophotometer, which has a maximum wavelength capacity of 730 nm, a wavelength of 700 nm was used for this study. For wavelengths that were essential for subsequent calculations but not directly measured (e.g. 473, 525 and 572 nm), interpolation was applied, as per AMSA [19] guidelines. For example, the reflectance at 473 nm was determined by interpolating between the recorded values at 470 and 480 nm. The initial reflectance values were then converted into reflex attenuation values (*A*), which represent the logarithmic inverse of reflectance. These values were subsequently used to calculate the percentage of MtMb content on the surface of raw beef patties, following the formula provided in AMSA [19] guidelines:

A=log1R,%OMb=2.375−A473700−AA525700−A×100,%MtMb=1.395−A572700−AA525700−A×100.

Here, *R* denotes the reflectance at a specific wavelength, *A* stands for the attenuance at that specific wavelength and OMb signifies oxymyoglobin and MtMb, the metmyoglobin content.

### 2.8. Cooking Yield and Texture Profiling Analysis

Each beef burger sample was preweighed, placed in a polypropylene zip bag and cooked in a water bath at 80°C for 50 min. After cooking, the samples were allowed to cool at room temperature for a few minutes. The accumulated water inside the polypropylene zip bag was drained, and the samples were gently blotted dry with absorbent paper before being reweighed. Cooking yield was calculated as a percentage using the following formula:

Cooking yield %=weight of cooked pattyweight of raw patty×100.

Texture profile analysis (TPA) was conducted to assess the hardness, springiness, cohesiveness, gumminess and chewiness of the beef patties, following the method described by Bambeni et al. [21] with minor modifications. Each sample was approximately 1 cm in height and was compressed twice to 75% of its original height using a circular flat‐surfaced disc with a 2 cm diameter. The test was performed in two compression cycles with a 1‐s rest period between cycles, using a TA1 texture analyser (Lloyd, Hampshire, United Kingdom) equipped with a 250 N load cell.

## 3. Statistical Analysis

Statistical analysis was conducted using SPSS Version 12.0 for Windows (IBM, SPSS Inc., Chicago, Illinois, United States) with the general linear model (GLM) procedure. Lipid oxidation, microbial growth, colour parameters, cooking yield and pH were analysed as dependent variables, whilst treatment was included as a between‐subject factor and storage time as a within‐subject (repeated measures) factor. Replicates were treated as random factors. Prior to analysis, the assumptions of normality and homogeneity of variance were tested using the Shapiro–Wilk and Levene′s tests, respectively. Microbial counts were log‐transformed where necessary to meet model assumptions. Significant differences between means were identified using Tukey′s post hoc test, with significance set at *p* < 0.05.

## 4. Results and Discussion

### 4.1. Antimicrobial Activities

**Figure 2:**
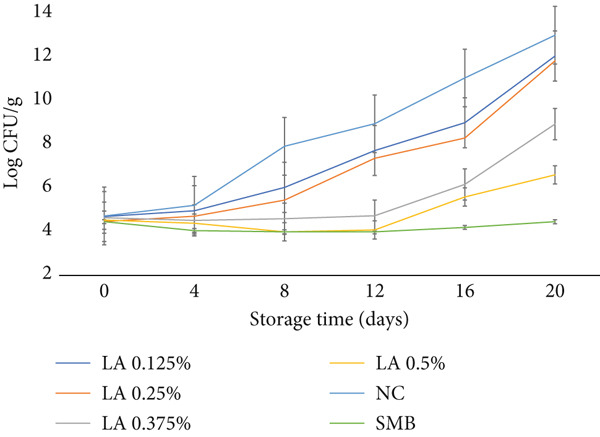
Total viable counts (TVC, log CFU g^−1^) of raw beef patties during storage (0, 4, 8, 12, 16 and 20 days). Treatments: NC (negative control), SMB (sodium metabisulphite, 450 ppm), LA (lactic acid) at 0.125%, 0.25%, 0.375% and 0.5% (w/w). Data are means ± SEM (*n* = 3 independent batches); error bars show standard error of the mean (SEM).

The TVC results are presented in Figure 2. Microbial growth was significantly influenced (*p* < 0.05) by the interaction between treatment and storage duration. Initially, no significant differences (*p* > 0.05) were observed between treatments. However, as storage progressed, microbial counts increased significantly (*p* < 0.05) over time across all treatments. The NC showed the highest counts by Day 20 (13 log CFU/g), whilst SMB‐treated samples consistently suppressed growth (< 5 log CFU/g). LA treatments delayed microbial proliferation in a dose‐dependent manner, with 0.5% LA showing the strongest inhibition compared with other LA levels (*p* < 0.05, T × S interaction). These findings align with previous research highlighting the strong antimicrobial properties of LA. For example, Stanojević‐Nikolić et al. [22] reported that LA exhibited broad‐spectrum antimicrobial activity against various strains, including both Gram‐positive and Gram‐negative bacteria, as well as fungi. Additionally, they found that LA was effective in reducing biofilm formation, a major concern in food processing environments. Similarly, Manzoor et al. [23] demonstrated that LA effectively inhibited microbial growth and extended the shelf life of buffalo meat.

The antimicrobial activity of LA observed in raw beef patties in this study can be attributed to its ability to lower pH, creating an acidic environment that inhibits microbial growth [24]. In addition to acidification, LA can disrupt bacterial cellular processes by interfering with energy metabolism, thereby inhibiting essential functions such as replication and protein synthesis [25]. Furthermore, LA destabilises the bacterial lipid bilayer, leading to increased membrane permeability, leakage of cellular contents and, ultimately, cell death [26]. These combined mechanisms highlight LA′s potential as an effective natural antimicrobial agent in meat preservation.

The strong antimicrobial effect observed in SMB‐treated beef patties can be attributed to its function as a bacteriostatic agent in meat systems [15]. SMB exerts its antimicrobial action by releasing sulphur dioxide (SO_2_) into the aqueous environment of the beef patty [27]. SO_2_ has broad‐spectrum antimicrobial properties and is highly toxic to many meat spoilage microorganisms. It disrupts cell membranes and depletes oxygen levels in the meat environment, thereby limiting the growth of aerobic microorganisms [28]. However, the use of SMB in meat products is strictly regulated in many countries and must comply with approved safety limits.

Additionally, some individuals may be sensitive or allergic to sulphites, experiencing adverse reactions such as headaches, nausea and respiratory difficulties [29]. These factors highlight the need for careful consideration when using SMB as a preservative in meat products.

### 4.2. Effect of LA on Lipid Oxidation in Raw Beef Patties

**Figure 3:**
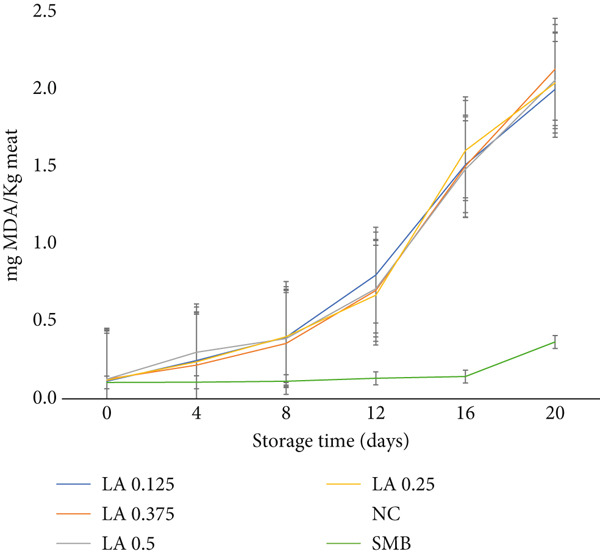
Lipid oxidation (TBARS, mg MDA kg^−1^) of raw beef patties during storage (0, 4, 8, 12, 16 and 20 days). Treatments: NC (negative control), SMB (sodium metabisulphite, 450 ppm), LA (lactic acid) at 0.125%, 0.25%, 0.375% and 0.5% (w/w). Data are means ± SEM (*n* = 3 independent batches); error bars show standard error of the mean (SEM).

Lipid oxidation levels were significantly influenced (*p* < 0.05) by the interaction between treatment and storage duration, as shown in Figure 3. However, no significant difference (*p* > 0.05) was observed between beef patties treated with LA and those without preservatives throughout the shelf‐life period.

This finding aligns with the study by Groussard et al. [30], which reported that LA did not affect lipid oxidation in a micelle model, suggesting that LA may not be effective in preventing lipid oxidation. However, other studies indicate that LA can function as an antioxidant through various mechanisms. For instance, LA has been shown to scavenge free radicals by donating a hydrogen atom [31], thereby neutralising free radicals and reducing oxidative damage. Nonetheless, further research is necessary to fully understand the potential benefits and limitations of using LA as an antioxidant in meat products.

Beef patties treated with SMB exhibited the lowest lipid oxidation levels throughout the study, with 0.37 mg MDA/kg of meat. In contrast, all LA‐treated and NC beef patties recorded lipid oxidation levels exceeding 2 mg MDA/kg by Day 20. Despite the effectiveness of SMB as an antioxidant in meat products, consumer concerns persist regarding the use of synthetic preservatives in food [32]. Therefore, it is essential to weigh the potential risks associated with SMB usage in meat products. Additionally, the study found that storage duration significantly influenced lipid oxidation levels (*p* < 0.05). This aligns with findings by Al‐Dalali et al. [16], who reported an increase in lipid oxidation in raw frozen beef as storage time progressed. These results highlight the importance of proper storage techniques to minimise lipid oxidation and maintain meat quality.

### 4.3. Effect of LA on the pH of Raw Beef Patties

**Figure 4:**
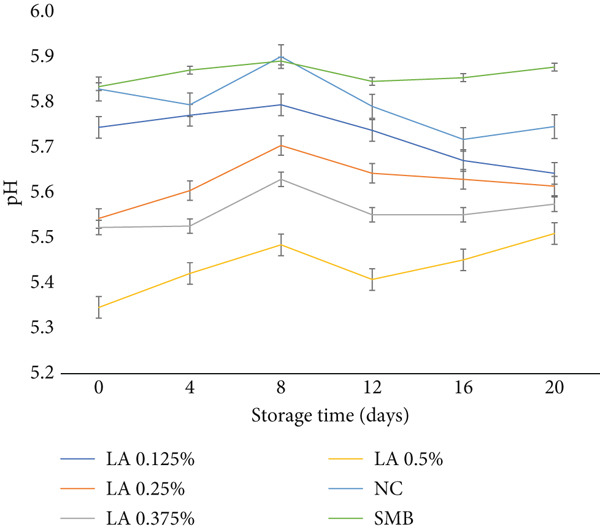
pH of raw beef patties during storage (0, 4, 8, 12, 16 and 20 days). Treatments: NC (negative control), SMB (sodium metabisulphite, 450 ppm), LA at 0.125%, 0.25%, 0.375% and 0.5% (w/w). Data are means ± SEM (*n* = 3 independent batches); error bars show standard error of the mean (SEM).

The pH results of the raw beef patties are shown in Figure 4. The study revealed that the treatment applied to raw beef patties significantly influenced pH values (*p* < 0.05). Specifically, beef patties treated with 0.125% or higher concentrations of LA exhibited lower pH levels compared to both the NC and SMB‐treated samples. Moreover, pH values in LA‐treated patties decreased progressively with increasing LA concentration. In contrast, storage time and the interaction between treatment and storage duration had no significant effect on pH values (*p* > 0.05), indicating that treatments maintained pH stability over time. The reduction in pH due to organic acids, including LA, occurs through dissociation [33]. Organic acids contain carboxyl groups that release hydrogen ions upon introduction into the meat system, thereby lowering the pH and increasing acidity [33].

### 4.4. Effects of LA on Colour Parameters of Raw Beef Patties

Colour is a primary quality attribute in fresh meat and one of the most critical factors influencing consumer purchasing decisions. Bright red colour is generally perceived as a marker of freshness, whilst discoloration, particularly browning associated with MtMb formation, signals spoilage and deters buyers. The results of the instrumental colour measurements are presented in Table 2. The *L*∗ value (lightness) of the beef patties was not significantly influenced (*p* > 0.05) by treatment, storage time or their interaction. However, the *a*∗ value (redness) was significantly affected by both LA treatment and storage time (*p* < 0.05), whilst their interaction had no significant effect (*p* > 0.05). Initially, all LA‐treated samples exhibited lower *a*∗ values compared to both the SMB‐treated and NC samples. As storage progressed (Days 4, 8, 12, 16 and 20), the *a*∗ value of NC beef patties declined more rapidly, reaching the lowest level amongst all samples, except for those treated with 0.5% LA. The lower *a*∗ values observed in LA‐treated samples during the early storage period may be attributed to their reduced pH. In minced meat systems, a lower pH accelerates the oxidation of ferrous myoglobin to ferric myoglobin, leading to discolouration. Additionally, reduced pH decreases the water‐holding capacity of meat, increasing surface moisture and light reflectance, which results in a paler appearance and reduced redness [34]. Over storage, the *a*∗ values of NC samples declined rapidly, consistent with oxidative discolouration, whilst higher LA concentrations (0.5%) partially mitigated this decline.

**Table 2:** Instrumental colour of raw beef patties containing either sodium metabisulphite or graded level of Davidson′s plum fruit powder.

**Attributes**	**Treatment (T)**	**Storage time (S) (days)**						**SEM**	**p** **value**		
		**0**	**4**	**8**	**12**	**16**	**20**		**T**	**S**	T*S
*L*∗	NC	46.4	44.1	45.9	42.0	46.9	47.2	0.94	0.17	0.13	0.54
	SMB	48.5	45.2	47.6	47.6	45.2	43.5				
	LA 0.125	48.3	45.6	43.1	44.7	45.6	45.3				
	LA 0.25	49.9	48.2	45.6	44.6	47.0	48.6				
	LA 0.375	46.3	45.0	46.2	45.3	44.3	46.3				
	LA 0.5	44.4	43.2	45.8	45.0	45.2	47.1				

*a*∗	NC	18.1^aA^	15.3^aAB^	12.6^aB^	12.7^aB^	8.5^bC^	7.2^cC^	0.52	< 0.001	> 0.001	0.082
	SMB	19.8^aA^	18.2^abA^	16.1^abAB^	16.1^abAB^	14.6^aB^	12.5^aB^				
	LA 0.125	15.4^bA^	15.9^abAB^	13.1^abABC^	11.3^bBC^	11.9^aC^	9.6^bC^				
	LA 0.25	15.1^bA^	13.1^bAB^	12.5^abAB^	11.3^bAB^	9.9^bB^	9.1^bB^				
	LA 0.375	15.3^bA^	13.2^bAB^	11.8^bBC^	11.5^bBC^	11.3^bBC^	9.1^bC^				
	LA 0.5	14.3^bA^	12.5^bAB^	9.7^bBC^	9.4^bBC^	9.1^bC^	8.3^bcC^				

*b*∗	NC	17.6^aA^	15.6AB	14.4^abBC^	12.4^bC^	12.7C	12.4C	0.36	< 0.001	> 0.001	0.054
	SMB	18.3^abA^	17.1^A^	15.8^aAB^	16.2^aA^	12.7^C^	12.9^BC^				
	LA 0.125	16.6^abA^	16.1^AB^	13.5^bBC^	13.3^bBC^	13.2^BC^	13.1^C^				
	LA 0.25	16.7^abA^	16.2^A^	15.8^aAB^	13.3^bC^	13.6^BC^	13.7^BC^				
	LA 0.375	16.1^abA^	15.3^AB^	14.6^abAB^	14.1^abAB^	14.1^AB^	13.5^B^				
	LA 0.5	15.1^bA^	14.6^AB^	13.5^bAB^	12.5^bB^	12.5^B^	12.9^AB^				

*H*∗	NC	44.1^A^	45.6^abC^	49.1^abBC^	44.7^bC^	56.3^AB^	59.5^A^	1.2	< 0.001	< 0.001	0.19
	SMB	43.1	43.1^b^	44.7^b^	45.9^ab^	48.8	49.5				
	LA 0.125	47.2	45.5^ab^	46.1^b^	49.3^ab^	48.4	54.4				
	LA 0.25	48.8	52.2^a^	51.6^ab^	49.8^ab^	54.4	56.4				
	LA 0.375	46.3^B^	49.2^abB^	51.6^abAB^	50.9^abAB^	51.1^AB^	56.7^A^				
	LA 0.5	46.8^C^	49.7^abBC^	55.3^aAB^	53.1^aABC^	54.1^ABC^	58.4^A^				

*C*∗	NC	25.3^abA^	21.9^abAB^	19.3^abcBC^	17.8^aCD^	15.4^DE^	14.5^E^	0.5	< 0.01	< 0.001	0.19
	SMB	27.1^aA^	24.9^aA^	22.5^aA^	22.9^bA^	17.4^B^	17.4^B^				
	LA 0.125	22.9^bcA^	22.6^abAB^	18.8^bcC^	17.5^aC^	17.9^C^	16.3^C^				
	LA 0.25	22.6^bcA^	20.9^bAB^	20.2^abABC^	17.5^aBCD^	17.1^CD^	16.6^D^				
	LA 0.375	22.2^bcA^	20.3^bAB^	18.9^bcBC^	18.3^aBC^	18.1^BC^	16.3^C^				
	LA 0.5	20.9^cA^	19.3^bAB^	16.7^cBC^	15.8^aC^	15.4^C^	15.4^C^				

*Note:* Results of instrumental colour measurement. The values are the means of triplicate samples. Not statistically significant (*p* > 0.05), statistically significant (*p* < 0.05). Means with different lowercase letters (a–c) in the same column are statistically different (*p* < 0.05) between treatments. Means with different uppercase letters (A–D) in the same row are statistically different (*p* < 0.05) over storage period.

Abbreviations: LA, lactic acid (0.125%, 0.25%, 0.375% and 0.5%); NC, negative control (450 ppm); S, storage period; SEM, standard error of the mean; SMB, sodium metabisulphite (450 ppm); T, treatments; T*S, interaction between treatment and storage period.

The *b*∗ and *H*∗ values also declined with storage (*p* < 0.05), indicating reduced yellowness and shifts in hue, whilst *C*∗ values were highest in SMB‐treated patties, suggesting a brighter and more vivid appearance. From a consumer perspective, colour is one of the most important quality attributes influencing purchase decisions, often serving as a visual indicator of freshness [35, 36]. The reduced redness and chroma observed in LA‐treated patties may negatively affect consumer perception of freshness and quality, despite LA′s natural preservative appeal. In contrast, SMB enhanced colour stability, but at the expense of consumer preference for clean‐label products [32]. These findings highlight the trade‐off between instrumental quality attributes and consumer acceptability, underlining the importance of balancing efficacy, visual quality and consumer expectations when selecting preservation strategies.

Finally, the *C*∗ value, which represents chroma or colour saturation [37], was significantly influenced (*p* < 0.05) by the interaction between treatment and storage time.

The LA‐treated beef patties showed no significant difference in *C*∗ values compared to the NC samples, whereas the SMB‐treated samples exhibited higher *C*∗ values. This suggests that SMB may have a greater impact on enhancing the colour saturation of beef patties than LA treatment. These findings underscore the importance of selecting appropriate treatments for meat products, as different preservatives can have varying effects on colour parameters, ultimately influencing the visual appeal and perceived quality of the product.

Numerous studies have demonstrated that consumers use colour as the primary visual cue when evaluating meat freshness and quality, often rejecting products with diminished redness or brown hues [36]. The lower redness and chroma values in LA‐treated patties, particularly at higher concentrations, may negatively influence consumer perception, despite LA′s appeal as a natural preservative. Whilst SMB clearly enhanced both redness stability and colour saturation, its application is increasingly restricted by regulatory limits and consumer demand for “clean‐label” alternatives. This trade‐off underscores the complexity of balancing technical efficacy with market acceptance.

The colour results highlight the limitations of LA as a stand‐alone preservative in minced beef systems, where acidification may exacerbate discolouration rather than prevent it. Conversely, SMB proved effective in maintaining colour stability but faces challenges in consumer acceptance. These outcomes emphasise the need for alternative strategies that combine LA with synergistic approaches such as MAP or natural antioxidants, thereby improving both visual quality and consumer acceptability in clean‐label meat products.

### 4.5. Effect of LA on the Surface Myoglobin Redox Forms

The surface myoglobin redox forms play a crucial role in determining the colour, flavour and overall quality of raw red meat products. The results of the surface myoglobin redox analysis are presented in Table 3. The OMb content was significantly influenced (*p* < 0.05) by the interaction between treatment and storage period. Initially, LA‐treated beef patties exhibited lower OMb values compared to both SMB‐treated and NC samples. However, as storage progressed, OMb levels in NC beef patties declined rapidly. By the end of the storage period, no significant difference in OMb content was observed between LA‐treated and NC samples. The initially lower OMb levels in LA‐treated samples may be attributed to the acidifying effect of LA, which lowered the pH and created a more acidic environment. pH fluctuations in meat systems can disrupt the redox balance of myoglobin, promoting the oxidation of ferrous myoglobin to MtMb (ferric myoglobin) [34]. This oxidation process likely contributed to the reduced *a*∗ values observed in LA‐treated raw beef patties.

**Table 3:** Surface myoglobin redox forms.

**Attributes**	**Treatment (T)**	**Storage time (S) (days)**						**SEM**	**p** **value**		
		**0**	**4**	**8**	**12**	**16**	**20**		**T**	**S**	T*S
OMb (%)	NC	83.7^aA^	82.4^aA^	82.9^abA^	82.9^abA^	71.2^abAB^	61.4^aB^	0.65	< 0.001	< 0.001	0.003
	SMB	88.4^a^	83.9^a^	88.7^a^	88.7^a^	86.1^aA^	86.5^b^				
	LA 0.125	82.6^abA^	81.6^aA^	79.7^abA^	79.7^aaA^	75.7^abAB^	68.5^aB^				
	LA 0.25	81.8^abA^	75.5^bAB^	76.0^bAB^	76.0^bAB^	74.1^abB^	70.6^aB^				
	LA 0.375	81.5^bA^	75.4^bB^	75.5^bB^	75.5^bB^	71.6^bBC^	66.3^aC^				
	LA 0.5	81.6^bA^	73.5^bA^	75.6^bA^	75.6^bA^	74.9^bA^	62.4^aB^				

MtMb (%)	NC	37.6^B^	39.2^bB^	40.4^cB^	40.4^bcB^	48.8^aA^	52.6^aA^	1.3	< 0.001	> 0.001	> 0.001
	SMB	37.5	38.3^bc^	38.2^a^	38.2^c^	38.7^b^	39.2^b^				
	LA 0.125	39.1^C^	40.2^bcC^	42.7^abBC^	42.7^abBC^	45.4^aAB^	50.2^aA^				
	LA 0.25	39.8^D^	43.5^aBC^	42.7^abCD^	42.7^abCD^	46.9^aAB^	49.6^aA^				
	LA 0.375	40.1^D^	43.5^abC^	45.7^aBC^	45.7^aBC^	46.6^aAB^	49.2^aA^				
	LA 0.5	41.2^C^	43.6^aBC^	46.2^aB^	46.2^aB^	47.1^aB^	52.8^aA^				

*Note:* Results of the surface myoglobin surface forms. The values are the means of triplicate samples. Not statistically significant (*p* > 0.05), statistically significant (*p* < 0.05). Means with different lowercase letters (a–c) in the same column are statistically different (*p* < 0.05) between treatments. Means with different uppercase letters (A–D) in the same row are statistically different (*p* < 0.05) over storage period.

Abbreviations: MtMb, metmyoglobin; NC, negative control; OMb, oxymyoglobin; SEM, standard error of the mean; SMB, sodium metabisulphite (450 ppm); SP, storage period; T, treatments; T*S, interaction between treatment and storage period.

Similarly, MtMb content in raw beef patties was significantly influenced (*p* < 0.05) by the interaction between treatment and storage period. During the initial stage of storage, LA‐treated beef patties exhibited higher MtMb content (*p* < 0.05) compared to SMB‐treated and NC samples. However, as storage progressed, MtMb levels increased significantly in all samples except those treated with SMB. By the end of the storage period, no significant difference in MtMb content was observed between LA‐treated and NC samples.

This suggests that the initially higher MtMb levels in LA‐treated patties may have resulted from the rapid oxidation of myoglobin to MtMb, facilitated by the acidic environment created by the lower pH [34].

Specifically, the reduced pH in LA‐treated patties may have induced myoglobin denaturation, accelerating the reaction between oxygen and the iron in the heme group, converting it from the ferrous (Fe^2+^) to the ferric (Fe^3+^) state and leading to the rapid formation of MtMb [38]. These findings align with the study by Poveda‐Arteaga et al. [39], which investigated intrinsic and extrinsic factors affecting MtMb production in red meats. Their research concluded that meat pH plays a crucial role, with lower pH accelerating myoglobin oxidation, ultimately leading to reduced redness in meat products.

### 4.6. Cooking Yield and Texture Profile Parameters

The TPA is a widely used method for evaluating the physical properties of food, including meat products such as beef patties. The results of the TPA, including springiness, hardness, cohesiveness, gumminess and chewiness, are presented in Table 4.

**Table 4:** Effect of lactic acid in raw beef patties on texture profile parameters and cooking yield.

**Attributes**	**Treatment**						**SEM**	**p** **values**
	**LA (0.125%)**	**LA (0.25%)**	**LA (0.375%)**	**LA (0.5%)**	**NC**	**SMB (450 ppm)**		
Springiness	0.87^abc^	0.87^ab^	0.83^bc^	0.79^c^	0.81^bc^	0.92^a^	0.22	< 0.001
Hardness (*N*)	107.6	114.7	118.3	107.8	107	98.5	5.6	> 0.05
Cohesiveness	0.44	0.45	0.47	0.46	0.44	0.45	0.022	> 0.05
Gumminess (*N*)	47.6	52.1	56.9	49.1	47	45	5.3	> 0.05
Chewiness (*N*)	36.9	45.7	45.8	38.9	38	40.9	5.2	> 0.05
Cook yield (%)	65.8	69.6	67.2	69.5	66.8	66.6	3.08	> 0.05

*Note:* Means for group in homogenous are displayed. NS (*p* > 0.05). Superscripts in the same row differ (*p* < 0.05). Means with different lowercase letters (a–c) in the same row differ (*p* < 0.05) between treatments.

Abbreviations: LA, lactic acid; NC, negative control; SEM, standard error of the mean; SMB, sodium metabisulphite.

The findings indicated no significant differences (*p* > 0.05) in most TPA parameters between treated and untreated beef patties. However, springiness was lower in LA‐treated samples. Springiness is a key attribute in meat products as it reflects elasticity [40]. A decrease in springiness suggests reduced elasticity in the beef patties, with values declining as LA concentration increased. This reduction may be attributed to protein denaturation caused by the acidic conditions introduced by LA [41]. Additionally, cooking yield results (Table 4) showed that LA treatment had no significant effect (*p* > 0.05) on the cooking yield of raw beef patties, indicating that LA did not impact moisture retention during cooking.

## 5. Conclusions

The use of LA as a natural preservative in raw beef patties demonstrated antimicrobial properties, effectively inhibiting the growth of undesirable bacteria. Although LA lowered the pH of the beef patties, it did not exhibit any antioxidant activity. The overall texture profile remained largely unaffected, except for a slight reduction in springiness. Notably, cooking loss was not influenced by LA addition. However, its application resulted in a decrease in the redness of the beef patties. These findings suggest that LA has potential as a natural preservative for beef patties and possibly other meat products. Nevertheless, further investigation is warranted to elucidate the underlying mechanisms of the observed effects and to comprehensively evaluate the influence of LA on the sensory characteristics of beef patties. Future research should also examine the potential of combining LA with natural antioxidants or other clean‐label interventions to enhance antioxidant activity and thereby improve product stability, colour preservation and overall quality.

## Disclosure

All authors have read and agreed to the published version of the manuscript.

## Conflicts of Interest

The authors declare no conflicts of interest.

## Author Contributions

Conceptualisation, planning and execution: M.M.B.; conceptualisation, review and editing: M.E.N., Y.S., H.E.S. and L.C.H.

## Funding

The authors genuinely thank the Australian Government for its financial support through the Australian Research Council′s Industrial Transformation Training Centre (ITTC) for Uniquely Australian Foods (Grant Number: IC180100045). Appreciation is also extended to Michel Beya′s PhD funding, provided by the Australian Government Research Training Program Scholarship. Additionally, the authors acknowledge the valuable support from Australian Country Choice (ACC), Earlee Products Pty Ltd and JBS Australia. Their contributions were instrumental in the successful completion of this research project.

## Data Availability

All data is in the manuscript.
